# Fluorescent Polyion Complex for the Detection of Sodium Dodecylbenzenesulfonate

**DOI:** 10.3390/polym10060657

**Published:** 2018-06-12

**Authors:** Shuai Liu, Cun Hu, Jianbin Huang, Yun Yan

**Affiliations:** 1 College of Chemistry and Chemical Engineering, Southwest Petroleum University, Chengdu 610500, China; shuailiu@swpu.edu.cn (S.L.); hucun402@stu.swpu.edu.cn (C.H.); 2 Beijing National Laboratory for Molecular Sciences, Institution College of Chemistry and Molecular Engineering, Peking University, Beijing 100871, China; jbhuang@pku.edu.cn

**Keywords:** polyion complexes, surfactant detection, competitive dissociation, HPTS, SDBS

## Abstract

Polyion complexes have been known about for decades, with their applications mainly restricted to drug and gene delivery. In this study, we show that by the introduction of fluorescent charged molecules into a polyion complex, it can be used as a specific detection system for surfactants. The fluorescence of 8-hydroxy-1,3,6-pyrenetrisulfonic acid trisodium salt (HPTS) is quenched in the ionic complex, while it can be recovered with the addition of the surfactant sodium dodecylbenzenesulfonate (SDBS), due to the stronger interaction between SDBS and the polyelectrolyte. This leads to a drastic color change of the solution, and a recovery of the strong emission of HPTS. Specifically, the fluorescence is linearly proportional to the concentration of SDBS, thus it can be used for the qualitative detection of SDBS. Furthermore, the detection limit for SDBS can be up to the order of 10^−10^ M. We believe that competitive dissociation of the ionic complex can be used as a general approach for the construction of new functional materials.

## 1. Introduction

Polyion complexes have attracted significant attention because of their facile formation [1,2,3,4] and great potential in biomedical applications [5,6], especially in drug delivery and gene transfection [7,8,9,10]. Diblock polyelectrolyte poly(*N*-methyl-2-vinylpyridinium iodide)-*b*-poly (ethylene oxide) (PMVP_41_-*b*-PEO_205_) has been widely employed to construct various polyion complexes with an oppositely charged component [11,12,13]. The polyion complexes can be made into micelles [14,15,16], vesicles [17,18,19], films [20,21], and even ultralong nanoladders [22]. So far, the practical application of various self-assemblies based on polyion complexes has mostly focused on drug and gene delivery, whereas other applications have not been sufficiently explored. This is mainly restricted by the fact that most of the self-assemblies are built with simple polyelectrolytes, which lack functional groups. In a previous work, the polyion complex built with PMVP_41_-*b*-PEO_205_ and 5,10,15,20-tetrakis (4-sulfonatophenyl) porphyrin (TPPS) was used to suppress the generation of singlet oxygen by TPPS [23]. Obviously, the employment of small charged functional molecules can endow polyion complex self-assemblies with rich functions. Since ionic complexation may change the physical properties of the encaged small molecules, we expect that sufficient competitive electrostatic interaction will allow dissociation of the ionic complex, thus triggering signal change. Clearly, this forms the basis for chemical sensing or detection. However, until now, insufficient attention has been paid to the use of polyion complexes in this manner, which is in great contrast to the increasing requirements for the precise and specific detection of chemicals in many fields.

It is well known that surfactants play an increasingly vital role in daily life, since they are extensively applied in domestic detergents, and in industrial and agricultural fields as emulsifiers, lubricants, thickening and foaming agents etc. [24,25]. As a result, surfactants have become one of the major pollutants in the natural environment. Thus, it is becoming important to be able to facilely detect trace amounts of surfactants in water. So far, many well known analytical methods, including high-performance liquid chromatography [26,27], GC/MS [28,29], potentiometry [30], capillary electrophoresis [31], flow injection analysis [32], and optical detection [33,34,35], have been developed for the detection of surfactants. However, all of these methods have limitations to some extent, such as time-consuming and tedious procedures, the requirement of expensive equipment, the use of toxic solvents, unsatisfactory selectivity, and sensitivity. In addition, not all surfactants do serious harm to the environment, as surfactants with aliphatic chains can be degraded by microorganisms within 1 week [36]. Only those with aromatic rings, such as sodium dodecylbenzenesulfonate (SDBS), which is the major component in detergent powders, produce notorious environmental problems. However, so far, an effective method for the specific detection of SDBS is still missing. Although high detection limits for SDBS have been reported with some designed small fluorescent molecules, or using conjugated polymers as probes [37,38,39,40,41], the detection of SDBS may be interfered with by the sodium salts of fatty acids. For this reason, concerns about the status of water are produced. Therefore, it is highly desirable to distinguish SDBS from other surfactants with aliphatic chains in a simple, selective, and sensitive way.

**Scheme 1:**
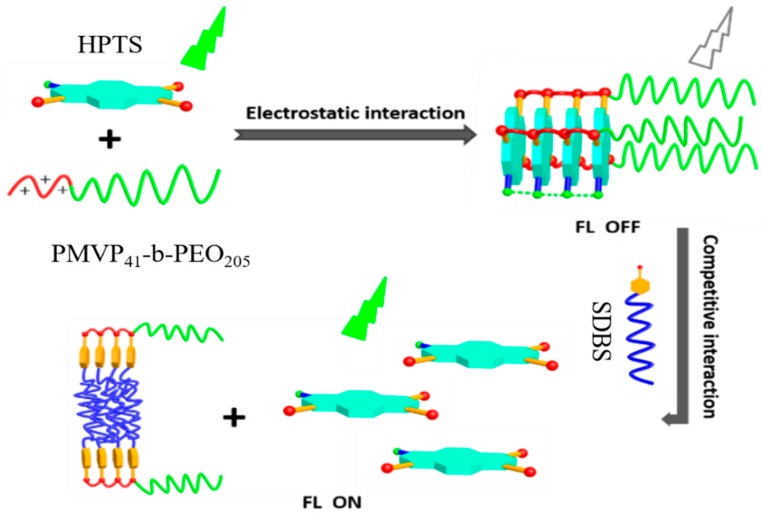
Illustration of the self-assembly of the HPTS/PMVP_41_-*b*-PEO_205_ complex and disassembly of the ionic self-assembled polyion complex in the presence of sodium dodecylbenzenesulfonate (SDBS).

In this work, we report that using the principle of competitive ionic interaction in a fluorescent polyion complex formed with a polymer containing the aromatic ring of pyridium, the facile visual specific detection of the surfactant sodium dodecylbenzenesulfonate (SDBS) is possible. The chemistry behind this is that the benzene ring in SDBS may specifically undergo aromatic interaction (π-π stacking) with the pyridium group, among other aliphatic surfactants. As a result, the release of the fluorescent species from the original ionic complex may be accompanied by the spectral change. To verify this hypothesis, we first built the ionic complex with PMVP_41_-*b*-PEO_205_ and the commercially available fluorescent molecule 8-hydroxy-1,3,6-pyrenetrisulfonic acid trisodium salt (HPTS). PMVP_41_-*b*-PEO_205_ is a cationic block copolymer containing pyridium groups, whereas HPTS carries three negative charges on a large aromatic plane, which allows it to efficiently form an ionic complex with the cationic block polyelectrolyte PMVP_41_-*b*-PEO_205_ through electrostatic and aromatic stacking interactions. Before complexation, HPTS displays significant fluorescence with a quantum yield (Ф) of 0.82 in water [42]. However, the emission of HPTS is quenched drastically upon the formation of an ionic complex with PMVP_41_-*b*-PEO_205_. We assume that the fluorescence of HPTS can be recovered by the introduction of SDBS, as opposed to other aliphatic surfactants, as SDBS offers additional aromatic interactions with the pyridinium group of the PMVP_41_-*b*-PEO_205_ when compared to other aliphatic surfactants. To this end, different anionic surfactants were added to the system, as they can interact with the cationic PMVP_41_-*b*-PEO_205_ through the synergistic effect of electrostatic interaction and hydrophobic interaction, thus disassembling the PMVP_41_-*b*-PEO_205_/HPTS complex. Indeed, the fluorescence of HPTS recovers with the addition of anionic surfactants. Remarkably, the extent of the fluorescence recovery strongly depends on the structure of the added anionic surfactant, and displays specific recovery behavior toward SDBS (Scheme 1). Meanwhile, the optical absorption also displays drastic changes upon the addition of SDBS. Therefore, we established a new colorimetric and fluorescent dual responsive system toward the specific detection of SDBS in water. To the best of our knowledge, this is the first time that the polyelectrolyte PMVP_41_-*b*-PEO_205_ has been used for the discrimination of a specific anionic surfactant.

## 2. Materials and Methods

### 2.1. Materials and Measurements

**Scheme 2:**
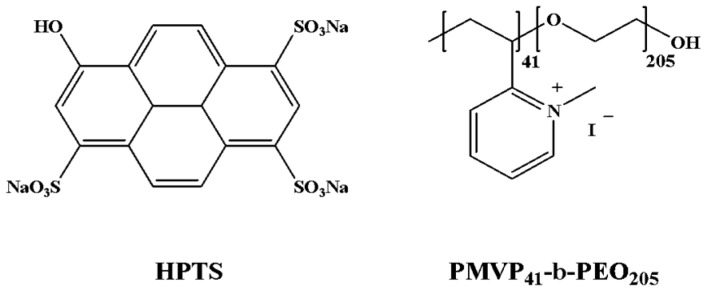
The molecular structures of 8-hydroxy-1,3,6-pyrenetrisulfonic acid trisodium salt (HPTS) and poly(*N*-methyl-2-vinylpyridinium iodide)-*b*-poly (ethylene oxide) (PMVP_41_-*b*-PEO_205_).

Diblock polyelectrolyte poly(*N*-methyl-2-vinylpyridinium iodide)-*b*-poly(ethylene oxide) (PMVP_41_-*b*-PEO_205_, *M*_w_ = 19 K, PDI = 1.05, about 90% quaternized) used in this work was prepared according to previously reported procedures [43]. 8-hydroxy-1,3,6-pyrenetrisulfonic acid trisodium salt (HPTS) was purchased from Sigma-Aldrich (Shanghai, China). The structures of HPTS and PMVP_41_-*b*-PEO_205_ are given in Scheme 2. All other chemicals were purchased from Beijing Chem. Reagents Co. (Beijing, China) and were used as received. Ultrapure water was used throughout the work. The other reagents were of A. R. grade.

A UV-1800 SHIMADZU spectrophotometer (Hitachi. Ltd., Tokyo, Japan) in the range of 200–700 nm was used to measure the absorption of solution samples.

A Hitachi F7000 Fluorescence Spectrometer (Hitachi. Ltd., Tokyo, Japan) was used to measure the fluorescence emission of solution samples. The excitation wavelength was set at 405 nm, which is the characteristic absorption peak of HPTS given in product information.

### 2.2. Sample Preparation

The PMVP_41_-*b*-PEO_205_/HPTS complex was prepared by adding PMVP_41_-*b*-PEO_205_ stock solution into a dilute aqueous solution of HPTS with the given concentration. Here, the concentration of PMVP_41_-*b*-PEO_205_ is the concentration of positive charges. The ratio of HPTS to PMVP_41_-*b*-PEO_205_ in the complex was kept at 1:8 for surfactant detection, since higher ratios lead to the waste of agents. All experiments were performed at room temperature (~298 K) unless otherwise specified.

## 3. Results and Discussion

### 3.1. The Formation of Polyion Complex with PMVP_41_-PEO_205_ and Fluorescent Molecule HPTS

**Figure 1:**
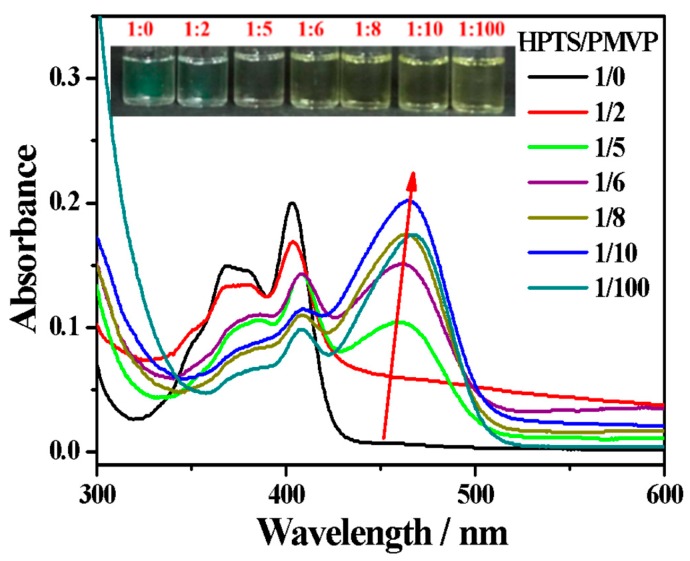
UV–Vis absorption spectra of 8-hydroxy-1,3,6-pyrenetrisulfonic acid trisodium salt (HPTS) (0.01 mM) in water at different charge ratios of HPTS: PMVP. Inset shows that the complex color with different charge ratios of HPTS: PMVP changes under daylight. PMVP is the abbreviation of PMVP_41_-*b*-PEO_205_ (poly(*N*-methyl-2-vinylpyridinium iodide)-*b*-poly(ethylene oxide)).

The cationic diblock polyelectrolyte poly(*N*-methyl-2-vinylpyridinium iodide)-*b*-poly(ethylene oxide) (PMVP_41_-*b*-PEO_205_) is chosen to construct a sensing system for surfactants, since it can easily form ionic self-assemblies with oppositely charged compounds. In this study, the negatively charged fluorescent molecule HPTS is employed to form an ionic complex with PMVP_41_-*b*-PEO_205_. Figure S1a shows that the absorption intensity increases with the increase in concentration of HPTS. The maximum absorption peak of HPTS is 403 nm. At the range of 0.001~0.1 mM, the intensity of the maximum absorption peak of HPTS increases in a linear fashion, as shown in Figure S1b, in line with the Lambert-Beer Law. Therefore, the HPTS exists as a monomer when the concentration is 0.01 mM. Figure 1 shows the absorption spectra of HPTS (0.01 mM) in aqueous solutions containing different concentrations of PMVP_41_-*b*-PEO_205_. HPTS on its own exhibits a characteristic absorption maximum at 403 nm. By increasing the concentration of PMVP_41_-*b*-PEO_205_, the absorption intensity at 403 nm gradually decreases, while the new absorption at longer wavelengths increases, and finally forms a major peak at 464 nm. In line with the occurrence of longer wave absorption, the solution displays a distinct color change from green to yellow (Figure 1. inset). Considering that the absorbance of PMVP_41_-*b*-PEO_205_ only occurs at 264 nm (Figure S2), the decrease of the absorbance at 403 nm and the appearance of a new band at 464 nm are ascribed to the formation of the polyion complex [44,45].

**Figure 2:**
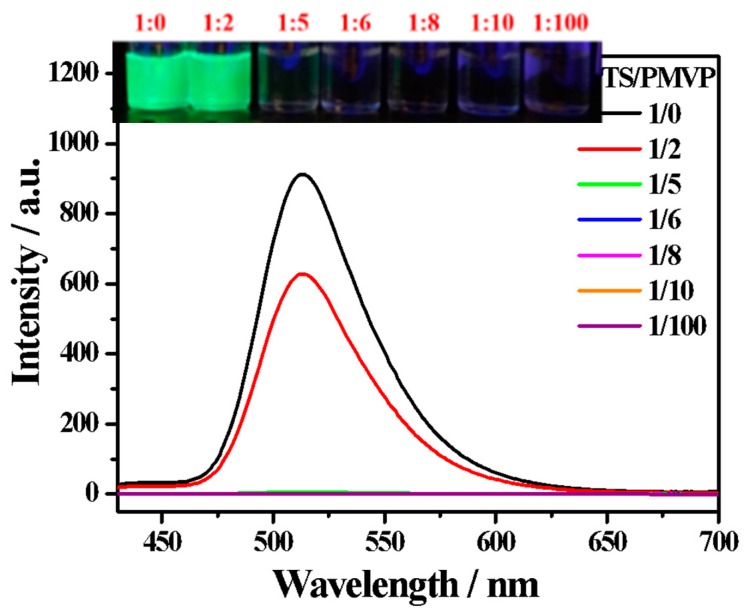
Fluorescence spectra of HPTS (0.01 mM) in water at different charge ratios of HPTS: PMVP. Excitation wavelength: 405 nm. Inset shows that the complex emission color with different charge ratios of HPTS:PMVP changes under a UV (365 nm) lamp. PMVP is the abbreviation of PMVP_41_-*b*-PEO_205_.

The fluorescence spectra of HPTS before and after the addition of various amounts of PMVP_41_-*b*-PEO_205_ are shown in Figure 2. Clearly, the emission maxima (512 nm) of the HPTS are gradually weakened by increasing the concentration of PMVP_41_-*b*-PEO_205_ in the solutions. The emission is completely quenched as the charge ratio of HPTS: PMVP_41_-*b*-PEO_205_ reaches 1:6. The photographs of these solutions under UV light (365 nm) reveal that the fluorescence quenching can be easily observed by the naked eye.

### 3.2. Detection of Anionic Surfactants Based on the Disassembly of the Ionic Self-Assembled Polyion Complex

Because the formation of the PMVP_41_-*b*-PEO_205_/HPTS complex is driven by electrostatic interaction, the optical properties of the ionic self-assembled complex are sensitive to any competitive ionic molecules. This provides us with an opportunity to detect molecules of interest in the solution. Since ionic surfactants are known to form well-defined ionic complex micelles with block polyelectrolytes [46,47,48], in the following paragraphs, we explore the possibility of detecting anionic surfactants using the present HPTS/ PMVP_41_-*b*-PEO_205_ system.

**Figure 3:**
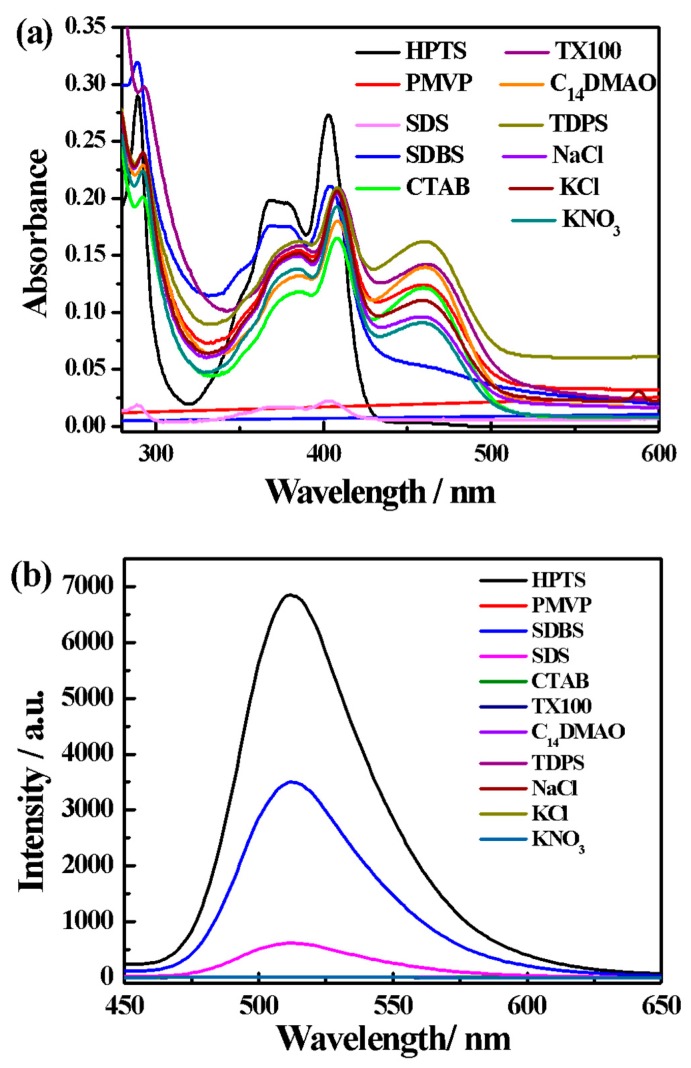
Absorption (**a**) and emission (**b**) spectra of the HPTS/PMVP_41_-*b*-PEO_205_ complex in water in the presence of various analytes. [HPTS] = 0.05 mM; [PMVP] = 0.4 mM; [SDS] = [SDBS] = [CTAB] = [Triton X-100] = [C_14_DMAO] = [TDPS] = 0.2 mM; [KCl] = [NaCl] = [KNO_3_] = 0.5 mM. PMVP is the abbreviation of PMVP_41_-*b*-PEO_205_.

To address the selectivity of the PMVP_41_-*b*-PEO_205_/HPTS complex toward anionic surfactants, various surfactants were added. Figure 3 shows the absorption and emission spectra of the PMVP_41_-*b*-PEO_205_/HPTS solution upon the addition of sodium dodecyl sulfonate (SDS), sodium dodecylbenzenesulfonate (SDBS), cetyltrimethylammonium bromide (CTAB), Triton X-100, tetradecyl dimethyl amine oxide (C_14_DMAO), tetradecyl dimethylammonium propane sulfonate (TDPS), and simple ions, including K^+^, Na^+^, and Cl^−^. Moreover, the absorption and emission spectra of the PMVP_41_-*b*-PEO_205_/HPTS solution upon the addition of the organic salts sodium salicylate (SS) and sodium benzoate (SB) are also measured, as shown in Figure S3a. Only anionic surfactants (SDS and SDBS) induce the distinct fluorescence recovery of HPTS at 512 nm, whereas the cationic (CTAB), non-ionic (C_14_DMAO), and zwitterionic (TDPS) surfactants and simple ions cannot recover the emission (Figure 3b). Similarly, the organic salts sodium salicylate (SS) and sodium benzoate (SB) are also unable to recover the emission (Figure S3b).

**Figure 4:**
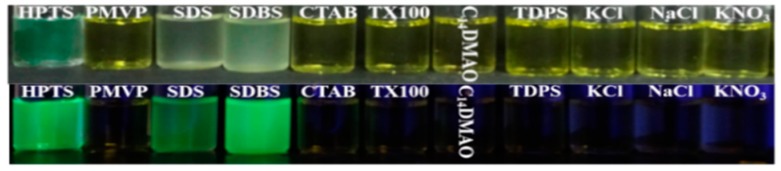
Changes in the color of HPTS/PMVP_41_-*b*-PEO_205_ complex in water in the presence of various analytes. [HPTS] = 0.05 mM; [PMVP-PEO] = 0.4 mM; [SDS] = [SDBS] = [CTAB] = [Triton X-100] = [C_14_DMAO] = [TDPS] = 0.2 mM; [KCl] = [NaCl] = [KNO_3_] = 0.5 mM. PMVP is the abbreviation of PMVP_41_-*b*-PEO_205._

Meanwhile, the optical appearance varies with the addition of different anionic surfactants. For instance, the system with SDBS is light green, while the system with SDS is pale yellow (Figure 4). In line with this, both the UV–Vis absorption and the fluorescence emission show significant spectral difference. The absorbance of HPTS at 403 nm increases, while the HPTS absorbance of the complex at 464 nm decreases upon the addition of SDBS, whereas the absorption triggered by SDS is drastically reduced. In addition, the emission with SDBS is an intense green, whereas the emission with SDS is light green. These drastic spectral differences allow us to distinguish between the anionic surfactants SDBS and SDS. Usually, it is hard to differentiate between anionic surfactants in an ionic complex, since they commonly display similar electrostatic interaction with polyions. It is possible that the benzene group in SDBS have interacted with the pyridine group in the PMVP_41_ block. Obviously, this specific interaction is much stronger than the nonspecific hydrophobic interaction that occurred between SDS and the PMVP_41_ block.

### 3.3. Distinction and Quantitative Detection of Sodium Dodecylbenzenesulfonate

**Figure 5:**
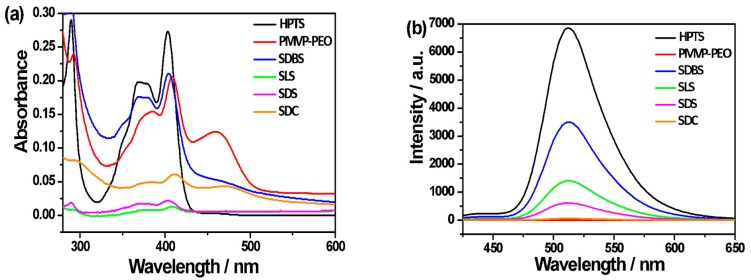
Absorption (**a**) and emission (**b**) spectra of the HPTS/PMVP_41_-*b*-PEO_205_ complex in water in the presence of different anionic surfactants. [PMVP-PEO] = 0.4 mM; [HPTS] = 0.05 mM; [SDBS] = [SLS] = [SDS] = [SDC] = 0.2 mM. PMVP-PEO is the abbreviation of PMVP_41_-*b*-PEO_205_.

To further confirm the specific detection of SDBS using the HPTS/PMVP_41_-*b*-PEO_205_ complex, different anionic surfactants were added into the HPTS/PMVP_41_-*b*-PEO_205_ solution. Figure 5a reveals that the characteristic absorption peak of HPTS at 403 nm can only be significantly recovered by the addition of SDBS. In contrast, this peak remains very low and broad as other surfactants are added. Accordingly, the emission spectra in Figure 5b manifest that only SDBS is able to induce the distinct fluorescence recovery of HPTS at 512 nm. Other anionic surfactants, such as sodium dichloroisocyanurate (SDC), SDS, and sodium lauryl sulfate (SLS), only trigger weak fluorescence recovery. Obviously, the HPTS/PMVP_41_-*b*-PEO_205_ ionic complex can specifically detect SDBS, mainly due to the strong affinity of sulfonate for the quaternary ammonium group [49,50].

**Figure 6:**
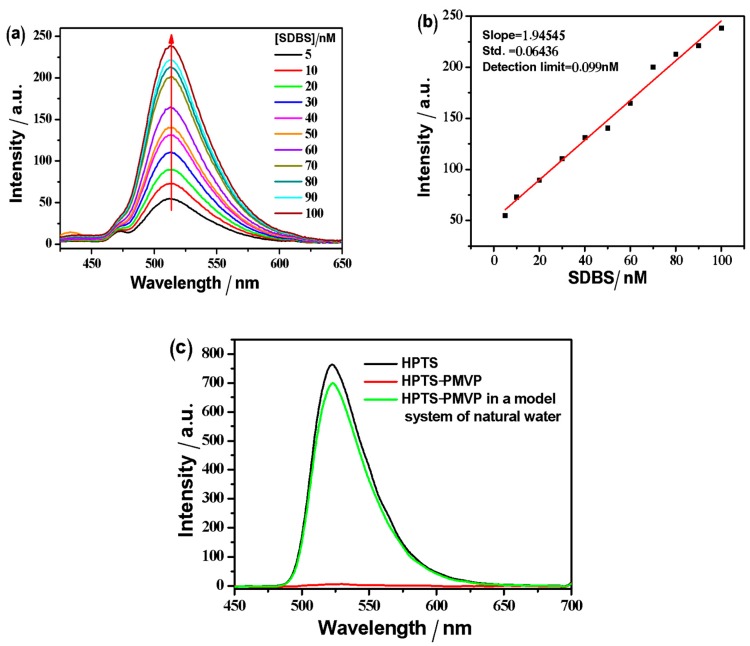
(**a**) Emission spectra of the HPTS/PMVP_41_-*b*-PEO_205_ complex in water in the presence of increasing amounts of SDBS; (**b**) The relationship between the emission intensity and the concentration of SDBS from 5 to 100 nM. R = 0.9903; (**c**) Emission spectra of the HPTS, HPTS/PMVP_41_-*b*-PEO_205_ complex and polyion complex in a model system of natural water which contains different surfactants, including SDS, SL, SDBS and simple organic ions. [SDBS] = 100 nM; [SDS] = [SLS] = 200 nM; [KCl] = [NaCl] = [KNO_3_] = 0.2 mM. PMVP is the abbreviation of PMVP_41_-*b*-PEO_205_. [PMVP_41_*-b*-PEO_205_] = 0.6 μM; [HPTS] = 0.1 μM. Excitation wavelength: 405 nm.

Considering the high quantum yield of HPTS, the establishment of a qualitative fluorometric analysis is anticipated by monitoring the emission of HPTS. Figure 6 displays the emission spectra of the PMVP_41_-*b*-PEO_205_/HPTS complex in aqueous solution in the presence of different amounts of SDBS. Without the presence of SDBS, the emission of HPTS is severely quenched. However, the emission intensity is gradually enhanced by increasing the concentration of SDBS. Notably, a good linear relationship exists between the emission intensity at 512 nm and the concentration of SDBS from 5–100 nM (R = 0.9903), with good reproducibility. This implies that the PMVP_41_-*b*-PEO_205_/HPTS complex is potentially useful for the quantitative determination of SDBS concentrations based on a fluorescence turn-on response of HPTS, and the detection limit can be up to the order of 10^−9^ M. From the slope of the fitting line of the data in the linear response range (*k*) and the deviation of fluorescent measurements (σ) [51], the detection limit of the fluorescent polyion complex towards SDBS (3σ/*k*) was calculated to be 0.1 nM. This high sensitivity toward SDBS is amazing; we attribute it to the additional aromatic interaction between the benzene ring in SDBS and the pyridium group of PMVP. This additional aromatic interaction also facilitates the practical application of the HTPS/PMVP_41_-*b*-PEO_205_ complex in the detection of SDBS in natural water. In Figure 6c, we show the experimental results of the detection effect in a model system of natural water containing different surfactants, including SDS, SL, and SDBS. We found that the fluorescence of HPTS is still observed, as the content of SDS and SL is twice as high as that of SDBS, confirming the practical possibilities.

## 4. Conclusions

In conclusion, the fluorescent ionic complex formed by PMVP_41_-*b*-PEO_205_ and HPTS can be employed in the quantitative detection of SDBS in water. The fluorescence of HPTS is quenched in the ionic complex, but can be recovered by the addition of SDBS. The stronger interaction between SDBS and PMVP_41_-*b*-PEO_205_ is responsible for this fluorescence recovery, which leads to the release of HPTS from the ionic complex. The release of HPTS is also accompanied by a significant change in the solution’s color, so that both the absorption and emission spectra can be applied to monitor the release of HPTS. We believe that the present work will not only develop a fast, simple, and convenient approach for sensing SDBS with the detection limit as low as 10^−9^ M, but will also provide an important clue to the construction of new functional materials based on polyion complexes.

## Supplementary Materials

The following are available online at http://www.mdpi.com/2073-4360/10/6/657/s1, Figure S1: (a) UV–Vis absorption spectra of HPTS solutions with a variety of concentrations. (b) The maximum absorption at 403 nm of HPTS solutions with a variety of concentrations; Figure S2: Absorption spectra of different concentrations of PMVP_41_-*b*-PEO_205_ in water; Figure S3: (a) Absorption and (b) Emission spectra of HPTS, HPTS/PMVP-PEO, HPTS/PMVP/SS, HPTS/PMVP/SB.
