# Supplementary material for: Fluorescent Polyion Complex for the Detection of Sodium Dodecylbenzenesulfonate
Source: Polymers (Basel). 2018 Jun 12;10(6):657. doi: 10.3390/polym10060657 (PMC6404151; doi:10.3390/polym10060657)
Supplement: Supplementary file 1 [file polymers-10-00657-s001.pdf]

Supplementary Materials

Article

Fluorescent Polyion Complex for Detection of Sodium Dodecylbenzenesulfonate

Shuai Liu [1], Cun Hu [1], Jianbin Huang [2] and Yun Yan [2,*]

1 College of Chemistry and Chemical Engineering, Southwest Petroleum University, Chengdu, 610500, China; 201599010093@swpu.edu.cn (S.L.); 201621000202@stu.swpu.edu.cn (C.H.)

2 Beijing National Laboratory for Molecular Sciences, Institution College of Chemistry and Molecular Engineering, Peking University, Beijing, 100871, China; jbhuang@pku.edu.cn (J.H.)

* Correspondence: yunyan@pku.edu.cn (Y.Y.); Tel.: 86-010-6276-5058

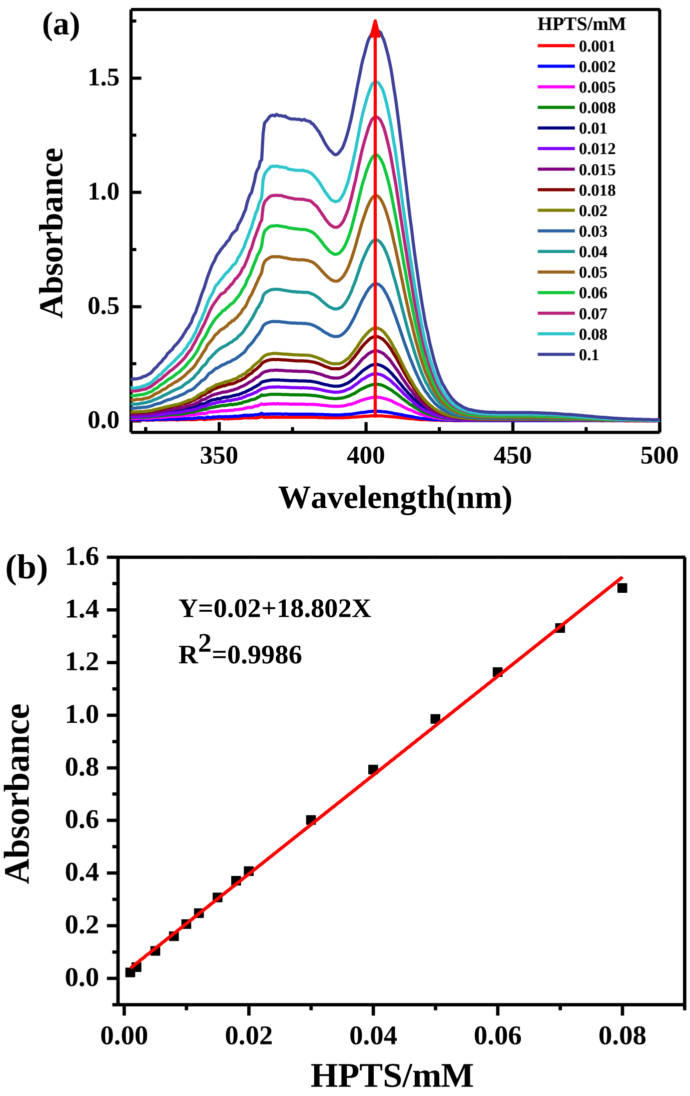

Figure S1. (a) UV-vis absorption spectra of HPTS solutions with varies concentration. (b) The maximum absorption at 403 nm of HPTS solutions with varies concentration. R=0.9986.

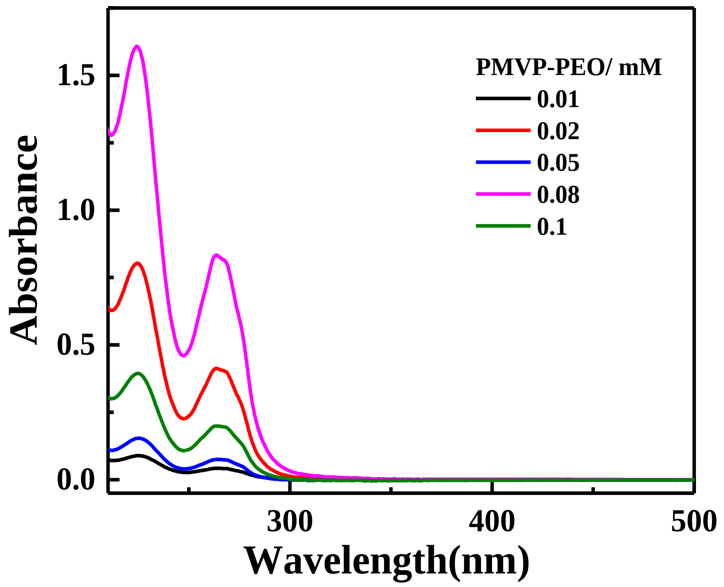

Figure S2. Absorption spectra of different concentrations $PMVP_{41}$-*b*-PEO_{205} in water. PMVP-PEO is the abbreviation of $PMVP_{41}$-*b*-PEO_{205}.

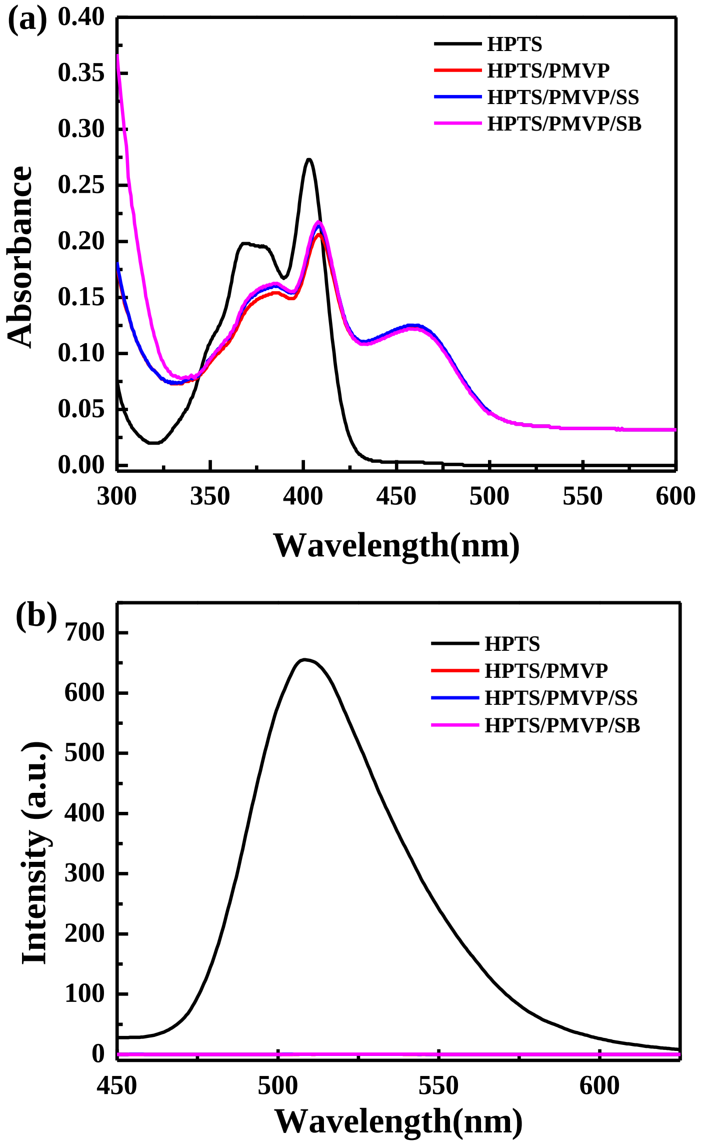

Figure S3. (a) Absorption and (b) Emission spectra of HPTS, HPTS/PMVP-PEO, HPTS/PMVP/SS, HPTS/PMVP/SB. [HPTS] = 0.05 mM, [PMVP] = 0.4 mM, [SS] = [SB] = 0.2 mM. PMVP, SS, SB is the abbreviation of $PMVP_{41}$-*b*-PEO_{205}, sodium salicylate, Sodium benzoate, respectively.
